# Analysis of the value of enhanced CT combined with texture analysis in the differential diagnosis of pulmonary sclerosing pneumocytoma and atypical peripheral lung cancer: a feasibility study

**DOI:** 10.1186/s12880-022-00745-1

**Published:** 2022-02-02

**Authors:** Chenglong Luo, Yiman Song, Yiyang Liu, Rui Wang, Jianbo Gao, Songwei Yue, Changmao Ding

**Affiliations:** Department of Radiology, The First Affiliated Hospital of Zhengzhou University, Zhengzhou, 450052 Henan Province China

**Keywords:** Computed tomography, Texture analysis, Pulmonary sclerosing pneumocytoma, Peripheral lung cancer

## Abstract

**Background:**

As a rare benign lung tumour, pulmonary sclerosing pneumocytoma (PSP) is often misdiagnosed as atypical peripheral lung cancer (APLC) on routine imaging examinations. This study explored the value of enhanced CT combined with texture analysis to differentiate between PSP and APLC.

**Methods:**

Forty-eight patients with PSP and fifty patients with APLC were retrospectively enrolled. The CT image features of the two groups of lesions were analysed, and MaZda software was used to evaluate the texture of CT venous phase thin-layer images. Independent sample t-test, Mann–Whitney U tests or χ^2^ tests were used to compare between groups. The intra-class correlation coefficient (ICC) was used to analyse the consistency of the selected texture parameters. Spearman correlation analysis was used to evaluate the differences in texture parameters between the two groups. Based on the statistically significant CT image features and CT texture parameters, the independent influencing factors between PSP and APLC were analysed by multivariate logistic regression. Extremely randomized trees (ERT) was used as the classifier to build models, and the models were evaluated by the five-fold cross-validation method.

**Results:**

Logistic regression analysis based on CT image features showed that calcification and arterial phase CT values were independent factors for distinguishing PSP from APLC. The results of logistic regression analysis based on CT texture parameters showed that WavEnHL_s-1 and Perc.01% were independent influencing factors to distinguish the two. Compared with the single-factor model (models A and B), the classification accuracy of the model based on image features combined with texture parameters was 0.84 ± 0.04, the AUC was 0.84 ± 0.03, and the sensitivity and specificity were 0.82 ± 0.13 and 0.87 ± 0.12, respectively.

**Conclusion:**

Enhanced CT combined with texture analysis showed good diagnostic value for distinguishing PSP and APLC, which may contribute to clinical decision-making and prognosis evaluation.

## Background

Pulmonary sclerosing pneumocytoma (PSP) is a rare pulmonary benign tumour originating from type II alveolar epithelial cells and was originally called sclerosing haemangioma. The WHO lung tumour classification 2015 version classified it as an "adenoma" and changed its name to PSP [1]. The clinical manifestations of PSP are nonspecific, and computer tomography (CT) images often appear as solitary nodules in the middle or outer zone of the lung field, which can show welt vessel signs, calcification, lobulation and so on [2, 3]. PSP lacks characteristic imaging findings, and without obvious malignant signs such as burr signs and pleural depression, it can easily be confused with atypical peripheral lung cancer (APLC) [4–6]. Clinically, surgical resection is the first choice for the treatment of PSP; there is no need for additional radiotherapy and chemotherapy, and the cure rate is good. However, the overall recovery of APLC is worse than that of PSP. Thus, careful evaluation and staging of the lesions are needed before surgical resection, and the accurate distinction between PSP and APLC before surgery is of great significance for the selection of appropriate surgery and treatment options.

At present, puncture biopsy and intraoperative rapid frozen pathology are important means to distinguish PSP from lung cancer. However, some studies have found it very challenging to distinguish PSP from lung cancer by needle biopsy or frozen section alone, and the misdiagnosis rate is quite high [7–9]. In addition, needle biopsy, as an invasive examination, carries the risk of bleeding, pneumothorax, and infection. Therefore, there is an urgent need to develop noninvasive auxiliary methods to accurately distinguish PSP from APLC before surgery to help pathologists and clinicians make an appropriate diagnosis and guide treatment strategies.

Texture analysis quantifies the abstract texture features in the image by analysing the grey levels of pixels, extracts detailed information that cannot be observed by the naked eye, and has a greater advantage in the identification of lesions with similar performance [10]. Based on a summary of the imaging characteristics of PSP and APLC, this study combined CT image texture analysis technologies to construct predictive models and analysed their value in differential diagnosis.

## Methods

Our retrospective study was approved by the institutional review board of the First Affiliated Hospital of Zhengzhou University. The requirement for informed consent from all patients was waived, and the whole study was performed by the World Medical Association guidelines and Declaration of Helsinki, revised in 2000 in Edinburgh.

### Patients

Patients with PSP and APLC confirmed by operation or puncture biopsy in the First Affiliated Hospital of Zhengzhou University from February 2016 to March 2021 were collected retrospectively. The inclusion criteria were as follows: (a) Before surgery or biopsy, both lung CT scans and dual-phase enhanced scans were available. The lesions were mainly solid, and the largest diameter of the lesions was less than 3 cm. (b) CT image quality met the requirements of texture analysis. (c) All cases were confirmed by histopathology. The exclusion criteria were as follows: (a) The lesion had received neoadjuvant radiotherapy and chemotherapy before CT examination. (b) Burr signs or pleural depression signs appeared in lung cancer lesions. (c) There was a history of other malignant tumours. Finally, 48 PSP patients were enrolled, including 13 males and 35 females, aged 22.0–76.0 years, with an average age of 50.7 ± 13.4 years, and 50 APLC patients (32 adenocarcinoma, 13 squamous cell carcinoma and 5 small cell carcinoma) were enrolled, including 31 males and 19 females, aged 34.0–83.0 years, with an average age of 63.2 ± 9.3 years.

### Imaging technique

All CT examinations were performed with Somatom Force (Siemens Healthineers, Erlangen, Germany). The scanning range was from the lung apex to the level of the adrenal glands on both sides. The CT scanning parameters were as follows: tube voltage 120 kVp; tube current 160 mA; pitch 1; field of view 350 mm × 350 mm; matrix 512 × 512; and slice thickness/interval 1 mm. The contrast agent for enhanced scanning was iodixanol (containing iodine 320 mg/ml), which was injected through the elbow vein at a flow rate of 3.5 ml/s according to the standard of 1.5 ml/kg. The arterial phase and venous phase scans were performed 35 s and 60 s after the injection of the contrast medium.

### Image feature analysis

Two radiologists with 5 and 8 years of experience in chest radiology retrospectively analysed the CT image performance and evaluated the signs. If there were disagreements, a senior chest radiology chief physician made the final judgement. The evaluation included the size (maximum diameter), shape (round, oval or irregular), plain scan CT value, enhanced scan CT value of the arterial phase and venous phase, degree of enhancement (mild, moderate, obvious enhancement), mode of enhancement (uniform or uneven), air gap, welt vessel sign, halo sign, calcification, lobular sign, liquefaction necrosis, and cavity. The evaluation criteria were as follows: (a) CT value: Measure the CT values of the solid components of the lesion in the plain scan, arterial phase and venous phase, avoiding blood vessels, calcification and liquefaction necrosis areas; each lesion was measured 3 times, and the average value was taken. (b) According to the increase in CT value, the degree of enhancement was divided into the following: obvious enhancement if the CT value increased by more than 40 Hounsfield units (HUs); moderate enhancement if the CT value increased by 20–40 HUs; and mild enhancement if the CT value increased by less than 20 HUs.

### Texture analysis and feature selection

**Fig. 1:**
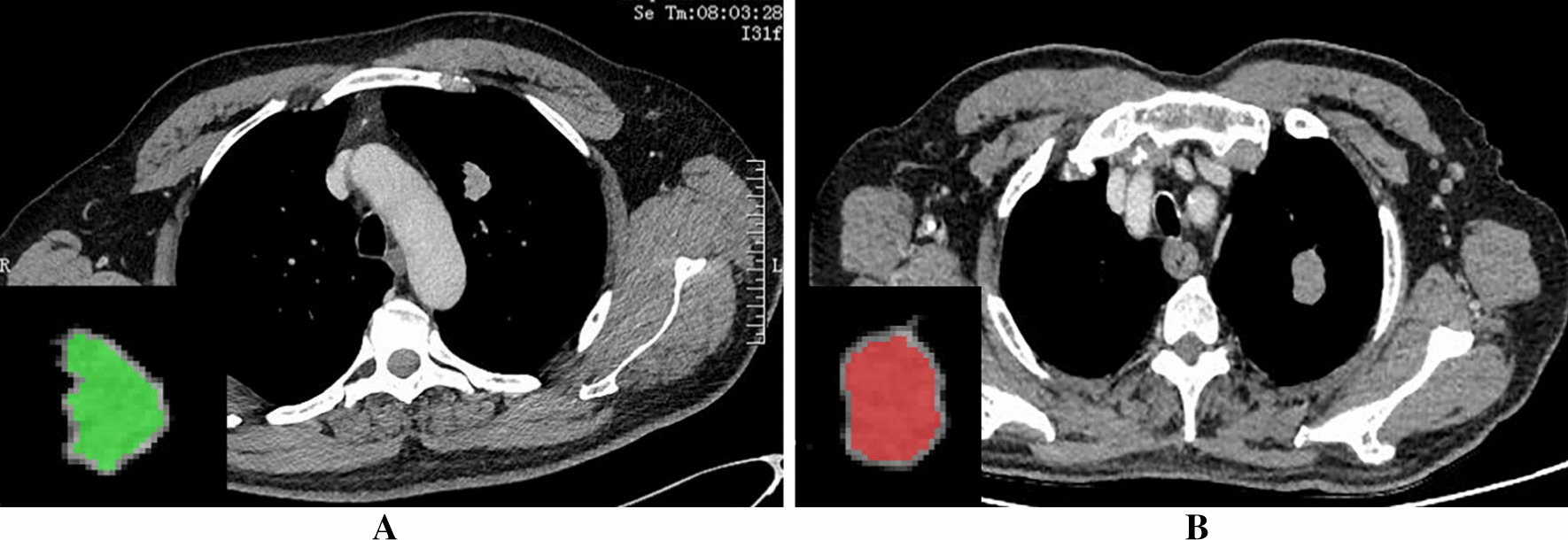
A 50-year-old man with a diagnosis of PSP (**A**). CT image of venous phase showed a left pulmonary nodule. The nodule is about 2.2 cm in length and diameter, with obvious uniform enhancement and lobulation sign at the edge. Manually outline the ROI 1-2 mm along the inner side of the nodule edge, which is indicated by the green area. A 76-year-old man with a diagnosis of Peripheral lung cancer (**B**). The CT image of the venous phase showed a left pulmonary nodule with a length of about 2.3 cm, moderately and uniformly enhanced, with smooth edges and lobular signs. Draw the ROI manually 1–2 mm along the inner side of the nodule edge, indicated by the red area

Compared with other stages, the lesions were shown more clearly in the venous phase. One study showed that during the intravenous phase of enhanced scanning, contrast medium can fill the microvessels in the lesions of lung cancer and better reflect the biological information and internal heterogeneity in the lesions [11]. Therefore, we selected the maximum cross section (window width 350 HUs, window level 40 HUs) of the lesions in CT venous phase thin slice images, and used MaZda software (Version 4.6, http://www.eletel.p.lodz.pl/mazda/) to analyse the texture. Image segmentation was performed by three radiologists using a double-blind method. The first segment was delineated by a radiologist (with 5 years of chest CT experience) and reviewed and revised by a senior chief physician (with 25 years of chest CT experience). In the second segment, a senior deputy chief physician (with 18 years of chest CT experience) drew the image alone. The detailed analysis process was as follows: (a) The selected image was imported into the software in BMP format, and μ ± 3SD (μ, grey-level mean; SD, standard deviation) was used for normalization of the image grey value to the maximum. Then, the influence of contrast and brightness on the grey value of the image was minimized. (b) The region of interest (ROI) along the inner side of the lesion edge 1–2 mm was manually outlined, and the PSP group and the APLC group were defined with two different colours (Fig. 1). (c) The software was used to calculate and generate 6 types of related texture parameters, including the grey-level histogram, grey-level cooccurrence matrix, grey-level run-length matrix, grey-level absolute gradient, autoregressive model and wavelet transform. Feature selection algorithms were included (Fisher coefficient [Fisher], mutual information [MI], probability of classification error and average correlation coefficient [POE + ACC]). The three texture feature selection algorithms were used to screen the best texture parameters.

### Statistical analysis

SPSS software (version 22.0, IBM Corp., Armonk, NY) was used for statistical analysis. The CT image features and the best texture parameters between the PSP group and APLC group were analysed by univariate analysis. Categorical variables were compared using Pearson’s chi-square test. Continuous variables were compared with an independent sample t-test or the Mann–Whitney U test if not normally distributed. The intra-class correlation coefficient was used to evaluate the consistency of texture parameters extracted by different testers. ICC > 0.75 was defined as consistent superiority. Spearman correlation analysis was used to evaluate the differences in texture parameters between groups, and to remove the redundant features with correlation > 0.9 to prevent over-fitting. Multifactor logistic regression analysis was used to analyse the remaining CT image features and CT texture parameters, and the independent factors between PSP and APLC were analysed. P < 0.05 was considered statistically significant.

### Model construction and evaluation

Extremely randomized trees (ERT) was used as the classifier to build the models based on the filtered CT image features and the CT texture parameters. The predictors included CT image features in Model A, CT texture parameters in Model B, and all image features and texture parameters in Model C. The models were verified by the five-fold cross-validation method to obtain stable results; that is, the original sample was divided into five subsamples of equal size. In the five subsample sets, a single subsample was retained as the verification data of the test model, and the remaining four subsamples were used as training data. Then, the cross-validation process was repeated five times, and the average values of five cross-validations were used. The prediction ability of the models was evaluated by classification accuracy, receiver operating characteristic (ROC) curves and areas under the curves (AUCs), sensitivity and specificity. All the above operations were implemented by Python v.3.7.9 (Python Software Foundation, DE, USA).

## Results

### Comparison of CT imaging features

The comparison results of CT imaging features of the two groups of lesions are shown in Table 1. The results suggest that PSP was mostly round or oval in shape, and internal calcification was more common, while the incidence of the lobular sign in APLC lesions was higher than that of PSP, and the above differences were statistically significant (P < 0.05). Compared with APLC, PSP lesions were mainly enhanced with a higher degree of enhancement; the difference in CT values between the two groups of lesions in the arterial and venous phases was statistically significant (P < 0.05). There were no statistically significant differences in the other CT imaging features between the groups (P > 0.05). Logistic regression analysis showed that calcification (odds ratio: 5.587, 95% confidence interval: 1.512–20.646, P = 0.010) and the CT value of the arterial phase (odds ratio: 0.949, 95% confidence interval: 0.925–0.975, P < 0.001) were independent influencing factors to distinguish the two.

**Table 1:** Comparison of CT image features between PSP and APLC

Imaging features	PSP (n = 48)	APLC (n = 50)	F-value (t/χ^2^)	*P-value*
Maximum diameter (cm)	1.9 ± 0.7	2.1 ± 0.6	-1.741^b^	0.085
Shape				
Round or oval	37	28	4.874^a^	0.027
Irregular	11	22		
Plain CT value (HU)	31.7 ± 11.6	30.4 ± 12.5	0.538^b^	0.592
CT value of the arterial phase (HU)	72.1 ± 20.7	53.4 ± 18.6	4.704^b^	< 0.001
CT value of the venous phase (HU)	76.9 ± 19.8	60.5 ± 17.9	4.284^b^	< 0.001
Degree of enhancement				
Mild	4	15	13.551^a^	0.001
Moderate	14	21		
Obvious	30	14		
Mode of enhancement				
Uniform	25	22	0.641^a^	0.423
Uneven	23	28		
Air gap	7	3	1.144^a^	0.285
Welt vessel sign	27	22	1.470^a^	0.225
Halo sign	6	5	0.154^a^	0.695
Calcification	14	4	7.318^a^	0.007
Lobular sign	10	24	7.977^a^	0.005
Liquefaction necrosis	4	7	0.789^a^	0.374
Cavity	1	4	0.760^a^	0.383

*PSP* pulmonary sclerosing pneumocytoma, *APLC* atypical peripheral lung cancer

^a^Pearson’s chi-square test

^b^Independent-sample t test

### Screening of CT texture parameters

MaZda software generated a total of 305 texture parameters, and the three feature extraction methods of Fisher, POE + ACC and MI each selected the 10 best texture parameters. Independent sample t-test or Mann–Whitney U tests showed that there were significant differences in 13 texture parameters between the two groups, and the ICC of these texture parameters in the reliability evaluation of the two segmentations was 0.790–0.997. After Spearman correlation analysis, 4 sets of texture parameters (Table 2) were reserved to construct the prediction models. Logistic regression analysis showed that WavEnHL_s-1 (odds ratio: 0.985, 95% confidence interval: 0.976 ~ 0.994, P = 0.002) and Perc.01% (odds ratio: 0.941, 95% confidence interval: 0.911 ~ 0.973, P < 0.001) were independent influencing factors to distinguish the two.

**Table 2:** Comparison of the best texture parameters between PSP and APLC

Texture parameters	PSP	APLC	Z-value /t-value	*P-value*
S(4,0)SumAverg^a^	81.0 ± 10.6	68.6 ± 9.2	5.693	< 0.001
Perc.01%^a^	125.8 ± 22.1	100.1 ± 17.2	6.260	< 0.001
WavEnHL_s1^b^	138.8 ± 183.2	54.5 ± 50.3	− 5.493	< 0.001
135dr_RLNonUni^b^	143.2 ± 281.7	362.6 ± 469.7	− 3.504	< 0.001

Continuous variables in the table expressed as means ± SD or medians ± IQR

^a^Means ± SD

^b^Medians ± IQR

### Model construction and evaluation

**Fig. 2:**
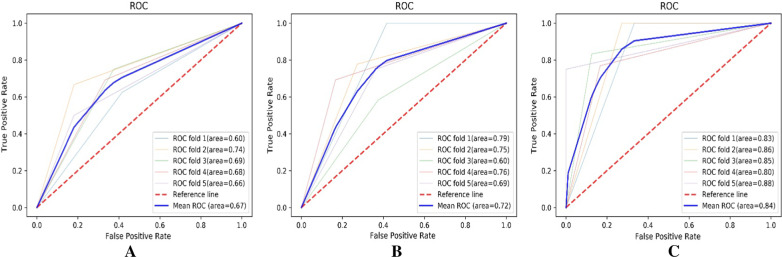
The ROC curves of five-fold cross-validation. Three models including CT image features (**A**), CT texture parameters (**B**), and CT image features combined with texture parameters (**C**) were used to distinguish PSP and APLC

Based on image features and texture parameters, prediction models were constructed by using the ERT classifier, and the models were validated by the five-fold cross-validation method. The effectiveness of each model to identify PSP and APLC is shown in Table 3 and Fig. 2. The model based on CT image features combined with texture parameters had the highest classification accuracy, which was 0.84 ± 0.04. The five-fold cross-validated AUCs of this model were 0.83, 0.86, 0.85, 0.80, and 0.88. The average AUC was 0.84 ± 0.03, the sensitivity was 0.82 ± 0.13, and the specificity was 0.87 ± 0.12.

**Table 3:** Comparison of the effectiveness of each model in identifying PSP and APLC

Model	AUC	Accuracy	Sensitivity	Specificity
Model A (CT image features)	0.67 ± 0.05	0.68 ± 0.05	0.70 ± 0.11	0.65 ± 0.09
Model B (CT texture parameters)	0.72 ± 0.08	0.70 ± 0.06	0.68 ± 0.10	0.76 ± 0.15
Model C (CT image features combined with texture parameters)	0.84 ± 0.03	0.84 ± 0.04	0.82 ± 0.13	0.87 ± 0.12

Continuous variables in the table expressed as means ± SD

*AUC* area under the curve

## Discussion

PSP is a rare benign lung tumour, accounting for approximately 2% to 3% of all lung tumours, and it is more common in women between 40 and 60 years old [12]. The clinical manifestations of the disease lack specificity. Some patients may present with cough, sputum expectoration, shortness of breath, haemoptysis, chest pain, and chest tightness, but these symptoms are of little clinical diagnostic value. PSP is composed of two basic types of tumour cells, namely, surface cells resembling type II pneumocytes and round cells; morphologically, PSP can be summarized into 4 characteristic areas (papillary area, solid area, sclerosis area, haemorrhage area), and the lesion is at least composed of two or more characteristic regions [13]. Pathological diagnosis of PSP before and during surgery is difficult, as some lesions need postoperative pathological examination and immunohistochemical analysis to make a definite diagnosis [9, 14]. Maleki et al. [8] believe that there is overlap between the morphological features of PSP and well-differentiated lung adenocarcinoma, so we should guard against the possibility of misdiagnosis. The complicated pathological structure of PSP determines the diversity of its imaging manifestations. Image signs such as regular morphology and calcification are more common in PSP, and similar manifestations can also appear in diseases such as lung cancer, hamartoma, inflammatory pseudotumours, and tuberculosis. Some PSPs also have imaging signs that tend to be malignant, such as lobulation and liquefaction necrosis, which are easily confused with APLC [15, 16]. Studies have found that the enhancement degree of lesions can distinguish PSP from other malignant tumours in the lung. In this study, the enhancement degree, arterial phase CT value and venous phase CT value of PSP were higher than those of APLC, and the difference was statistically significant, which is consistent with the above research results [17]. The distinction between PSP and APLC based on the abovementioned imaging signs is currently the main diagnostic mode, but the subjective analytical process of the physician is often affected by various factors, such as the size of the lesion, personal experience, and image quality. Shin et al. [15] retrospectively analysed CT images of 76 patients with PSP, and the accuracy of CT diagnosis before pathological confirmation was only 30.3%. In this study, the accuracy of model A based on CT image features for distinguishing PSP from APLC was 0.68 ± 0.05, and the AUC was 0.67 ± 0.05. There is still room for improvement in the accuracy of traditional imaging features to distinguish between the two.

Different from subjective image feature analysis, CT texture analysis can extract a variety of feature parameters from the image texture that cannot be distinguished by the naked eye and quantitatively analyse the heterogeneity of lesion tissue structure, and tumour heterogeneity is one of the important features that distinguishes malignant tumours from normal tissues or benign lesions [18]. Studies have shown that texture analysis based on CT images has good application prospects for the diagnosis of benign and malignant pulmonary lesions, pathological classification and staging of lung cancer [19–21]. The ERT is an integrated machine learning algorithm, and its base estimator is a decision tree. Different from the random forest algorithm, ERT uses all the samples for training and randomly extracts features so that the ERT bifurcates the attributes completely randomly, and the splitting process does not prune until a decision tree is generated. ERT can effectively reduce the deviation and variance of sample data, and has powerful generalization ability [22]. This study is based on ERT classifier modelling, and aims to analyse the differential diagnostic value of enhanced CT image features, texture analysis, and the combination of the two in distinguishing PSP and APLC.

In this study, logistic regression analysis based on CT texture parameters showed that WavEnHL_s-1 and Perc.01% were independent influencing factors to distinguish PSP from APLC. Perc.01% is derived from the grey-level histogram, which indicates the average grey value within a 1% voxel interval from the left side of the histogram. Yue et al. [23] conducted a CT texture analysis study and found that compared with colorectal adenocarcinoma, Perc.01% is more common in signet-ring cell carcinoma, which may be related to the tiny necrotic area in signet-ring cell carcinoma lesions. In this study, the Perc.01% value of PSP was higher than that of APLC, and the difference was statistically significant, which may indicate that there were relatively fewer areas of haemorrhage and necrosis with low grey values ​​in PSP lesions. WavEnHL_s-1 is derived from wavelet transform, which can detect possible structures in the image, especially fine structural details that cannot be detected by the naked eye. Features based on wavelet transform can amplify the subtle changes in intensity between regions and represent the unevenness of intensity within the region. The larger the value is, the more uneven the density of the CT image [24]. Zhang et al. [25] performed a radiomics study of CT images and reported that WavEnLH_s-3 is an independent influencing factor to distinguish focal organizing pneumonia from peripheral lung adenocarcinoma. In this study, the WavEnLH_s-1 value of PSP was higher than that of APLC, and the difference was statistically significant, suggesting that there may be a large difference in the density uniformity of CT images between the two groups. Compared with model A, the classification accuracy of model B based on CT texture parameters was improved slightly to 0.70 ± 0.06.

Traditional imaging examination combined with texture analysis has advantages in identifying difficult lesions. Zhang et al. [26] further improved the differential diagnostic ability of mass-forming pancreatitis and cancer in the pancreatic head by constructing an enhanced CT combined texture analysis model. In this study, the classification accuracy of model C based on image features combined with texture parameters was significantly improved, which was 0.84 ± 0.04. This result was similar to the above study, suggesting that enhanced CT combined with texture analysis may be a new auxiliary method to distinguish PSP from APLC before surgery.

There are several limitations in this study. First, this is a feasibility study on the identification of PSP and APLC with enhanced CT combined with texture analysis. The sample size is limited. In the future, cooperation with other medical centres is needed to expand the sample size and conduct external verification. Second, using manual methods to outline ROIs, there is a certain degree of subjectivity and nonreplicability. Third, we only performed texture analysis on the largest layer of the axial image of the lesion, and did not analyse all layers of the tumour. However, a study showed that two-dimensional texture analysis gave sufficient results, although multi-slice volume analysis may be more representative of tumours [27]. We believe that it is more convenient to use the two-dimensional level to perform texture analysis, which is conducive to extensive clinical applications. Finally, the prediction model constructed in this study is only applicable when PSP and APLC are highly suspected in clinical practice, and the clinical application scenarios are limited. Next, we will increase the applicability of the model by including more types of lung masses.

## Conclusions

Enhanced CT combined with texture analysis had good diagnostic value for distinguishing PSP and APLC and could become a useful supplement to routine imaging and pathological diagnosis.

## Data Availability

The raw data cannot be made freely available because of privacy restrictions but the datasets used and/or analysed during the current study available from the corresponding author on reasonable request (dcm526@126.com).

## Abbreviations

CT: Computed tomography
PSP: Pulmonary sclerosing pneumocytoma
APLC: Atypical peripheral lung cancer
ICC: Intra-class correlation coefficient
ERT: Extremely randomized trees
HUs: Hounsfield units
ROC: Receiver operating characteristic
AUCs: Area under the curves
ROI: Regions of interest
Fisher: Fisher coefficient
MI: Mutual information
POE + ACC: Probability of classification error and average correlation coefficient
μ: Gray-level mean
SD: Standard deviation
IQR: Indicates interquartile range
